# Supplementary material for: Chromothripsis during telomere crisis is independent of NHEJ, and consistent with a replicative origin
Source: Genome Res. 2019 May;29(5):737–49. doi: 10.1101/gr.240705.118 (PMC6499312; doi:10.1101/gr.240705.118)
Supplement: Supplemental Material [file supp_gr.240705.118_Supplemental_file_1.zip › contigs/annotated_contigs/DB48/contig.2.DB48_length_347_mean_cov_5.95389048991.docx]

**DB48_length_347_mean_cov_5.95389048991**

```
AAATCTGTCACTACAATTTAAACACCAGTCCCGAAATTTGGATCTTCTTTCTTTTTGAATCTCTCAAACGGCAACATTCCTCAGAAACC
 >chr7:50469169-50469391 + E=2e-122
AAAGCTTTATTTCAAATCTCTTCCTTCCCTGGCTGGTTCCATCTAGTACCAGAGGCCTCTTTTCCTGAAGAAATCCAATCCTAGCCCTC

ATTTTAATTATGTACATCTGTTTGTAGCCACAAGCCTGAATT|TC|AAGACTGGGACTTAGAATTCCCACGTCCAGCACTGTTGTAGAT
                                           >chr7:50472250-50472377 - E=5e-65
GGGAGGTACGTTGTGCTGAACTGCTGCGCTGGCCACATCAAGACACCACATGTGTTCCCAAGTGGTATTGCAGCTGTCCTGA
```
